# Biostimulants promote the accumulation of carbohydrates and biosynthesis of anthocyanins in ‘Yinhongli’ plum

**DOI:** 10.3389/fpls.2022.1074965

**Published:** 2023-01-06

**Authors:** Lu Yao, Dong Liang, Hui Xia, Yazhuo Pang, Qiao Xiao, Yan Huang, Wen Zhang, Changbing Pu, Jin Wang, Xiulan Lv

**Affiliations:** College of Horticulture, Sichuan Agricultural University, Chengdu, China

**Keywords:** biostimulants, anthocyanins, carbohydrates, Plum (*Prunus salicina* L.), Principal component analysis (PCA)

## Abstract

Biostimulants play an important role in promoting crop growth and development and improving fruit yield, but their influence on fruit quality in horticulture plants is still unclear. In this study, four types of biostimulants, Ainuo (AN), Aigefu (AG), Weiguo (WG), and Guanwu Shuang (GS) were applied to the fruit surface of ‘Yinhongli’ plum at 60 and 75 days after anthesis to investigate their effect on carbohydrates and biosynthesis of anthocyanins, and also analyze the relationship between sugar and anthocyanin accumulation during fruit color change to ripening. Results showed that all biostimulant treatments significantly improved fruit appearance quality, and increased single fruit weight and TSS/TA. Cyanidin 3-*O*-glucoside and cyanidin 3-*O*-rutinoside, are the most important anthocyanins in the red skin of the ‘Yinhongli’ plum, and no anthocyanin was detected in the green skin. In addition, WG and GS treatments significantly increased the expression of structural genes involved in anthocyanin biosynthesis compared with the control, especially *chalcone synthase (CHS)* and *flavonoid 3-O-glucosyltransferase (UFGT)* at 95-105 d after anthesis, leading to anthocyanin accumulation 10 days earlier than the control. Correlation analysis showed that there was a significant correlation between total sugar and anthocyanin content during fruit coloring and ripening.

## 1 Introduction

Plum (*Prunus domestica* Lindl.) is highly favored by consumers due to its various varieties, colors, fresh taste, rich nutrition, and natural antioxidant functional components (Roussos et al., 2016; Liu et al., 2020). Fruit variety and yield are important factors affecting the fruit quality (Tomić et al., 2019; Hamdani et al., 2022). Chinese plums (*Prunus salicina* Lindl) and European plums (*Prunus domestica* Lindl) are the main production cultivars of plums. Compared with European plums, which are mostly used for processing, Chinese plums are mainly used for fresh food due to their crispier and sweeter flesh. Plum fruits have red, green, yellow, and purplish-black, which greatly contributes to the visual quality of the fruits. Anthocyanins are the main pigments responsible for plums, and usually give bright colors to fruit (Jaakola, 2013; Ge et al., 2022). Anthocyanins, one kind of flavonoid, are products from the phenylpropanoid biosynthesis pathway, which have very important nutritional and pharmacological values (Kalt et al., 2019; Ghattamaneni et al, 2019).

The anthocyanin biosynthesis pathway has been elucidated in a variety of plant species, such as apples (Li et al., 2022), peaches (Khan et al, 2021), and strawberries (Shen et al., 2020). Numerous studies have shown that anthocyanin accumulation is influenced by complex interactions of environmental and developmental factors, such as light, temperature, and the level of sugar, and plant hormones (Nardozza et al., 2019; Fang et al., 2021; Gao et al., 2021). Previous studies found that hot air and UV-C treatments promoted the synthesis of anthocyanins and PAs by enhancing the activities and expressions of enzymes involved in phenylpropanoid metabolism during peach fruit development (Zhou et al., 2020a). Fulvic acid applied to the foliage or soil of ‘Flordaprince’ peach trees significantly increased fruit yield, fruit size, soluble solids content, and anthocyanin concentration in the pericarp (EI-Razek et al, 2012). Carbohydrates, especially sugars, also induce anthocyanin accumulation by interacting with MBW complex genes in plant species (Payyavula et al., 2013). Sugars (glucose, fructose, sucrose) serve both as the main precursor for the biosynthesis of phenylpropanoids and as signal substances that regulate their synthesis (Das et al., 2012). However, Different kinds of sugar in the biosynthesis of anthocyanins in plums are still unknown.

‘Yinhongli’ plum, belonging to the Chinese plum, is a local variety of Yibin City, Sichuan province, with crisp and sweet flesh and easily separated stone. Unlike other plum varieties, the peel of ‘Yinghongli’ is only partially red and mostly green when ripe. Importantly, the size of the red area of the peel directly affects the perception of the fruit and the selling price. Consumers prefer fruit with the more red area because it has higher bioactive substances (Gramza-Michałowska et al., 2019; Chen et al., 2021). Therefore, some agronomic practices have been attempted to improve fruit quality and coloration, including the spraying of biostimulants (Soppelsa et al., 2019; Francesca et al., 2020; Distefano et al., 2022). Biostimulants are natural substances, according to the officially definition of the EU (EU, 2019), that can enhance the absorption and utilization of nutrients by stimulating the natural physiological process of plants, including humic acid, seaweed extract, protein hydrolysate, amino acids, chitosan and its derivatives, microbial and bacterial preparations, etc. (Battacharyya et al., 2015). In recent years, biostimulants have been reported to promote plant vigor (Dong et al., 2020), as well as strawberry fruit quality (Weber et al., 2018; Rahman et al., 2018), apricot (Tarantino et al., 2018), and sweet cherry (Gonçalves et al., 2020). However, the application of biostimulants in other horticultural crops has not been seldom studied. In this study, four types of biostimulants were applied to ‘Yinhong’ plum’ to investigate their effects on fruit quality and anthocyanins accumulation. The results will lay the foundation for further studies on the regulation mechanism of sugar on anthocyanin accumulation in ‘Yinhongli’ plum.

## 2 Materials and methods

### 2.1 Plant material and treatments

The field experimental were carried out in an orchard in Yibin city, Sichuan Province, China (28°28’N, 28°54’E). The altitude is about 800 meters, the annual average temperature is 14.9 ℃, the sunshine hours are 970 hours, and the frost-free period is more than 300 days. The soil had a pH of 4.4, organic matter of 3.7%, total nitrogen (N) of 1.42g kg^−1^, total phosphorus (P) of 12 g kg^−1^, available P of 1.6 mg kg^−1^, and available K of 48 mg kg^−1^. Ten-year-old ‘Yinhongli’ plums were randomly selected for treatments. Four biostimulants products, Ainuo (AN, humic acid ≥30g/L, North China Pharmaceutical Group Ainuo Co., Ltd.), Aigefu (AF, seaweed extract ≥20g/L, Cape South Africa Co., Ltd.), Weiguo (WG, amino acids ≥100g/L, Guangdong Weisheng greenhouse Technology Co., Ltd.), and Guanwu Shuang (GS, amino acids ≥110g/L, Syngenta China Investment Co., Ltd.), were sprayed on the leaves and fruits of ‘Yinhongli’ plum at 60 d and 75 d after anthesis. The recommended concentration of the products was used, and the trees that were not sprayed were used as controls (CK). Each treatment was designed with three replications and three trees per replicate.

At least 27 fruits were collected for each treatment at 60 d, 75 d, 80 d, 90 d, and 105 d (ripening) after anthesis (DAA), respectively. All samples were brought back to the laboratory in ice boxes as soon as possible. After single fruit weight, longitudinal, transverse diameter and color, peel and flesh were separatedand frozen with liquid nitrogen, then stored at −80 °C.

### 2.2 Determination of fruit quality parameters

Ten fruits were randomly selected for each treatment, and the color of each fruit was measured on both symmetrical sides at the equator (mid-latitude line) using a colorimeter CR-10 (Konica Minolta, Tokyo, Japan) as described previously (Mcguire, 1992). Fruit firmness was determined using a GY-4 digital fruit sclerometer (GY-4; Jinyang, Beijing, China), and total soluble solids were quantified using a digital refractometer (ARP-TD32, Airui pu, China). The soluble sugar content, and titratable acid content (in terms of malic acid) of the fruit were determined by the method of Dong et al. (2002). All index measurements were designed with three biological repetitions.

### 2.3 Determination of anthocyanin content

Total anthocyanin content was detected according to the method described by Olivares et al. (2017). About 1 g Frozen skin samples were ground into fine powder in liquid nitrogen, extracted with methanol: HCl (99:1, v/v), and ultrasonic extraction was carried out at 40°C for 60 min. The extract was centrifuged at 8000×g for 15 min. The pH of the supernatant was adjusted to 1 or 4.5. The absorbance was measured at 520 nm and 700 nm, respectively.

The anthocyanin components of skin were extracted according to the method of Chen et al. (2007) with slight modification. Under dark conditions, 0.5 g of frozen red and green skin samples were ground on ice, dissolved in 1.5 mL of 1% HCl-methanol, mixed until homogenized, and extracted at 4°C for 12 h. The extracts were centrifuged at 10,000 × g at 4 ℃ for 10 min, and the supernatant was filtered with a microporous membrane (0.45μm) for high-performance liquid chromatography (HPLC, Agilent 1260 Series) analysis, and Comatex C18 column (250 mm x 4.6 mm, 5 μm, Plainfield, USA), column temperature 30 ℃. The elution system consisted of a mobile phase A (ultra-pure water) with a gradient elution procedure at a flow rate of 1.0 mL·min^-1^, injection volume of 10.0 μL, column temperature of 30 ℃, and detection wavelength of 520 nm. Anthocyanin components were identified by comparison the peak with of standard sample cyanidin 3-*O*-glucoside (Cy3G) and cyanidin 3-*O*-rutinoside(Cy3R, bought from Solarbio, Beijing, China). Quantification of anthocyanin was performed based on the peak area of the sample recorded at 520 nm.

### 2.4 Determination of the content of glucose, fructose, sucrose, and sorbitol

The extraction and determination of sucrose, glucose, fructose, and sorbitol were carried out according to the methods of Wu et al. (2007), with slight modifications. 1.0 g of the sample was added to 4 mL of ultrapure water, and heated at 80 °C for 15 min. After centrifugation and filtering, the supernatant was analyzed on an Agilent 1260 HPLC system (Agilent Technologies), equipped with a Thermo NH2 column (4.6 mm ×250 mm, 5 μm, Angela Technologies, Shanghai, China). The mobile phase consisted of acetonitrile/water (80:20, v/v)) at a flow rate of 1.0 mL/min, and the column temperature was 40°C.

### 2.5 Principal component analysis and comprehensive evaluation

The data were analyzed using SPSS 26.0 for principal component analysis (PCA) to build the model.

The PCA reduces the complexity of the data and finds the most important features. Assuming that there are n samples in a practical problem. Each sample has p indexes which are regarded as p random variables and recorded as X1, X2 … Xp. Through PCA, p indicators are standardized, reduced dimensions, performed linear regression and formed k principal components F1, F2 … Fk (k ≤p) according to equation. The scores of different treatment samples were calculated and ranked to evaluate the sensory quality.

### 2.6 Gene expression analysis in skin by quantitative RT-PCR (qRT-PCR)

Candidate gene sequences related to anthocyanin synthesis and sugar metabolism were searched fome Genbank. for in the ‘Sanyueli’ plum and Prunus domestica gene sequence libraries using the TBtools tool, and BLAST (https://blast.ncbi.nlm.nih.gov/Blast.cgi) for comparison (details are shown in **Table S1**).

Total RNA was extracted using the RNA prep Pure polysaccharide polyphenol plant total RNA (TIANGEN biotech, Beijing, China) and the Prime ScriptTM RT reagent Kit with gDNA Eraser (Perfect Real Time) kit (TaKara, Dalian, China) for reverse transcription. All steps were performed according to the reagent instructions. Quantitative PCR was conducted using the TB Green^®^ Premix Ex Taq™ II (Tli RNaseH Plus) (TaKara, Dalian, China). The reaction procedure was 95 ℃ for 30 s, 95 ℃ for 5 s, and TM for 30 s for 40 cycles. Each treatment was designed with three biological repetitions. The melting curve was analyzed, and the relative expression levels of each gene were calculated by the 2^-ΔΔCt^ method.

### 2.7 Statistical analysis

All values are mean ± SD of three biological repeats. Duncan’s method was used to test the significance using SPSS 26.0 (SPSS, Inc., Chicago, IL, USA). Figures were elaborated using SigmaPlot 14.0 (Systat, San Jose, CA, USA).

## 3 Results

### 3.1 Effect of biostimulants on fruit quality

Compared to the control (CK), the application of biostimulants differentially stimulated the fruit growth (**Table 1**), with the increase of fruit weight in treatments of AN, WG and GS at ripening (105 DAA), accompanied with no significant change of firmness. AN, AG and GS treatments increased TSS, the WG and GS treatments decreased TA content, resulting in improvement of TSS/TA ratio in all treatments. The sugar/acid ratio is an important indicator determining fruit quality.

**Table 1:** Effects of biostimulants on the fruit appearance quality.

Treatment	Fruit weight (g/fruit)	Firmness (kg.cm^−2^)	TSS (%)	TA (%)	TSS/TA
CK	45.88 ± 2.91b	16.90 ± 1.21ab	9.41 ± 0.07c	0.87 ± 0.03a	10.80 ± 0.37c
AN	51.30 ± 1.76a	19.14 ± 2.48a	11.83 ± 0.06b	0.79 ± 0.06ab	15.11 ± 1.13b
AG	45.29 ± 2.74b	17.28 ± 2.36ab	12.15 ± 0.28ab	0.77 ± 0.05ab	15.78 ± 1.01ab
WG	51.43 ± 2.38a	13.54 ± 1.30ab	9.72 ± 0.12c	0.69 ± 0.03c	14.03 ± 0.42b
GS	54.17 ± 1.80a	14.42 ± 2.22b	12.37 ± 0.24a	0.67 ± 0.08d	18.79 ± 2.68a

Values are mean ± SD (n=10). Different letters indicate significantlences among treatments at *p*< 0.05 by one-way ANOVA test. Treatments: CK, control; AN, humic acid; AF, seaweed extract; WG, seaweed extract; GS, amino acid.

### 3.2 Effects of biostimulants on the coloration and anthocyanins content

**Figure 1:**
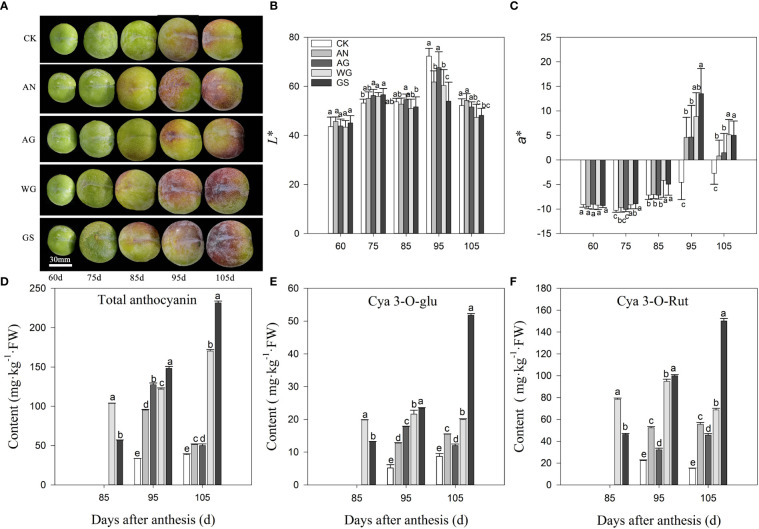
Effect of biostimulants on skin color **(A)**, L* value **(B)**, a* value **(C)**, total anthocyanin content **(D)**, and contents of anthocyanin components **(E, F)**. Values are mean ± SD. (n =3). Different letters indicate significantly different values at *p*< 0.05.

After biostimulant application, the skin color of ‘Yinhongli’ plum turned light red at 85 DAA, while the control treatment appeared green. The skin turned color which was 10 d earlier than that of CK (**Figure 1A**). Correspondingly, the a* values of all treatments were significantly higher than that of CK at 95 DAA and 105 DAA (**Figures 1B, C**). Application of biostimulants significantly increased the content of total anthocyanins and anthocyanin components in the skin (**Figures 1D–F**). Total anthocyanins content was almost undetectable in the skin of ‘Yinhongli’ plum at 85 DAA. During fruit ripening, the total anthocyanin content in CK, WG and GS treatments reached the peak at 95 DAA and then decreased. WG and GS treatments reached the peak at 105 DAA, which were much higher than that in other treatments. The main anthocyanin component in red skin detected was cyanidin 3-*O*-rutinoside, followed by cyanidin 3-*O*-glucoside, and no anthocyanin was tested in the green skin. At 105 DAA (fruit ripening stage), the GS treatment had the maximum content of anthocyanin, with 51.79 mg·kg^-1^ FW cyanidin 3-*O*-glucoside and 150.1 mg·kg^-1^ FW cyanidin 3-*O*-rutinoside, which was significantly higher than the other treatments.

### 3.3 Effect of biostimulants on fruit carbohydrates

**Figure 2:**
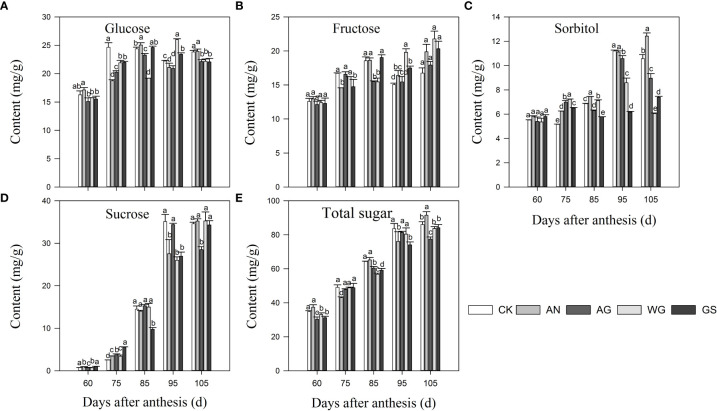
Effects of biostimulants on the content of glucose **(A)**, fructose **(B)**, sorbitol **(C)**, sucrose **(D)**, and total sugar **(E)**. Values are mean ± SD (n =3). Different letters indicate significantly different values at *p*< 0.05.

Four soluble sugars, glucose, fructose, sucrose, and sorbitol, were separated from the pulp of the ‘Yinhongli’ plum (**Figure 2**). The highest sucrose content was found in ‘Yinhongli’ plum fruits at maturity, followed by glucose and fructose. Glucose and fructose accumulated rapidly from young fruits to green fruits at 60 d and 75 DAA. At 85-95 DAA, there was a significant decrease in glucose and fructose in CK, AG, WG, and GS treatments, while sorbitol content accumulated substantially at this time. At 105 DAA, the sorbitol content decreased significantly. The content of sorbitol was consistently lower during the fruit ripening. At 105 DAA, the fructose content was higher in AW and AX treatments compared to other treatments, which were 20.32 mg/g and 21.78 mg/g, respectively. Sucrose contents in all treatments fruit increased throughout the ripening process of the ‘Yinhongli’ plum and were higher than other soluble sugars at 105 DAA, indicating that the ‘Yinhongli’ plum belongs to the sucrose accumulation type.

### 3.4 Evaluation of fruit quality based on PCA

The eigenvalues and contribution rates of the principal components (PC) are the basis of selecting the PCs; the larger the eigenvalues, the more information the PC contains. In this experiment, PCA was performed with 11 measured variables (Fruit weight, Firmness, TSS/TA, L*, a*, cyanidin 3-*O*-rutinoside, cyanidin 3-*O*-glucoside, total anthocyanins, glucose, fructose, sorbitol, Sucrose, and total sugar). The variance contribution rate of PC of ‘Yinhongli’ plum fruit quality variation showed in **Tables 2**-**4**. The first three PCs gave eigenvalues greater than 1.0 and explain 85.73% of the total variation. As shown in **Table 3**, the contribution rate of the first PC accounted for 58.61% of the total variance. Fruit weight (X1), TSS/TA (X3), a* (X5), cyanidin 3-*O*-glucoside (X6), cyanidin 3-*O*-rutinoside (X7), total anthocyanins (X8), Fructose (X10) and Sucrose (X12) primarily reflected PC1 with contributing rates of 0.763, 0.823, 0.936, 0.880, 0.932, 0.947, 0.735 and 0.076, respectively. These variables are important indexes to evaluate the fruit quality of ‘Yinhongli’ plum. The second PC explained 27.91% of the total variation, with Firmness (X2), Sucrose (X12) and total sugar (X13) contributed 0.857, 0.928, and 0.948, respectively. The results indicated that the sugar of the ‘Yinhongli’ plum is the main information source of PC2. The contribution rate of a* (X5) is 0.070, which is the color index of sugar content, representing 8.65% of the total variation. The scores of the three PCs showed that the score of GS treatment was the highest (0.60), followed by AN (0.35) and WG (0.29), indicating the higher comprehensive quality of the GS treatment.

**Table 2:** Eigen values and contribution rates of principal components.

Component	Initial eigenvalues	Contribution rate of variance (%)	Cumulative variance contribution rate (%)
*1*	7.620	58.613	58.613
2	3.628	27.909	86.522
*3*	1.124	8.645	95.167
4	.628	4.833	100.000
5	5.240E-16	4.031E-15	100.000
6	3.423E-16	2.633E-15	100.000
7	1.597E-16	1.229E-15	100.000
8	-2.422E-17	-1.863E-16	100.000
9	-9.772E-17	-7.517E-16	100.000
10	-2.172E-16	-1.670E-15	100.000
11	-3.930E-16	-3.023E-15	100.000
12	-4.510E-16	-3.470E-15	100.000
13	-6.237E-16	-4.798E-15	100.000

**Table 3:** Component matrix.

Parameters	Contribution rate		
	PC1	PC2	PC3
Fruit weight(X1)	0.763	0.638	0.100
Firmness(X2)	-0.410	0.857	-0.210
TSS/TA(X3)	0.823	-0.091	0.519
L*(X4)	-0.979	0.001	0.202
a*(X5)	0.936	-0.012	0.070
cyanidin 3-*O*-glucoside(X6)	0.880	0.161	0.314
cyanidin 3-*O*-rutinoside(X7)	0.932	0.151	0.298
Total anthocyanins(X8)	0.947	0.168	-0.137
Glucose(X9)	-0.797	0.561	0.208
Fructose(X10)	0.735	0.486	-0.300
Sorbitol(X11)	-0.754	0.299	0.567
Sucrose(X12)	0.076	0.928	-0.243
Total sugar(X13)	-0.258	0.948	0.187

PC, principal component; X1….X13, 13 random variables. L*represents the relative brightness, a*represents the balance between green and red.

**Table 4:** Principal component scores after standardization.

Sample	F1	F2	F3	F	Rank
CK	-1.02	-0.11	-0.23	-0.68	5
AN	-0.27	1.71	0.16	0.35	2
AG	0.14	-2.23	0.15	-0.55	4
WG	0.58	0.08	-1.05	0.29	3
GS	0.56	0.55	0.97	0.60	1

### 3.5 Effect of biostimulants on glucose metabolism genes

**Figure 3:**
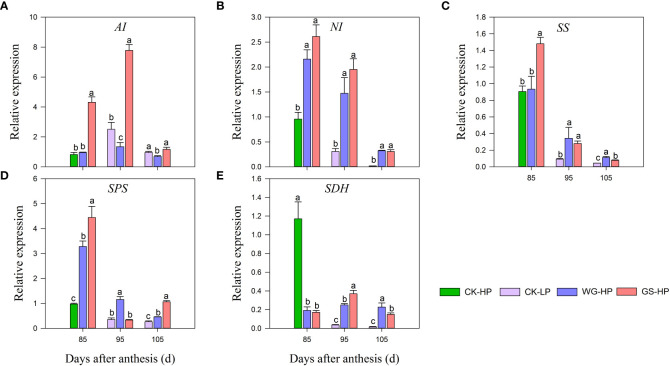
Transcript levels of sugar-related genes in different treatments. CK-LP means green skin of ‘Yinhongli’plum, the rest are red skin. Values are mean ± SD (n =3). Different letters indicate significantly different values at *p*< 0.05. AI, acid invertase **(A)**; NI, neutral invertase **(B)**; SS, sucrose synthase **(C)**; SPS, sucrose phosphate synthase **(D)**; SDH, Succinate dehydrogenase **(E)**.

qRT-PCR was performed to determine the expression levels of genes involved in sugar metabolic, such as *acid invertase (AI), neutral invertase (NI), sucrose synthase (SS), sucrose phosphate synthase (SPS)*, and *Succinate dehydrogenase (SDH)* (**Figure 3**). During the fruit ripening, a continuous decrease in the transcript level was observed in *NI, SS*, and *SPS* in CK, with low expression occurring at the fruit ripening stage. While after treatments all structural genes were significantly up-regulated compared with CK. The high expression of *SS* and *SPS* transcript levels of structural genes at the early stage of coloration promoted the accumulation of sucrose content, which was consistent with the rapid increase of sucrose content at 85-95 DAA. The opposite was true for glucose and fructose contents (**Figure 2**), indicating that this is the stage of rapid sucrose accumulation and the decomposition rate of sucrose reduces. The expression levels of *SS* and *SPS* decreased late in fruit development, sucrose was partially broken down into glucose and fructose, and glucose and fructose content started to increase. Upon control treatment, the expression of sugar metabolism structural genes *AI* and *SDH* peaked at 85 DAA (only green skin expression) and continued to down-regulate expression after coloration. WG and GS-treated red skin first increased and then decreased, but overall *SDH* transcript levels were lower.

### 3.6 Effect of biostimulants on genes expression involved in anthocyanin biosynthesis

**Figure 4:**
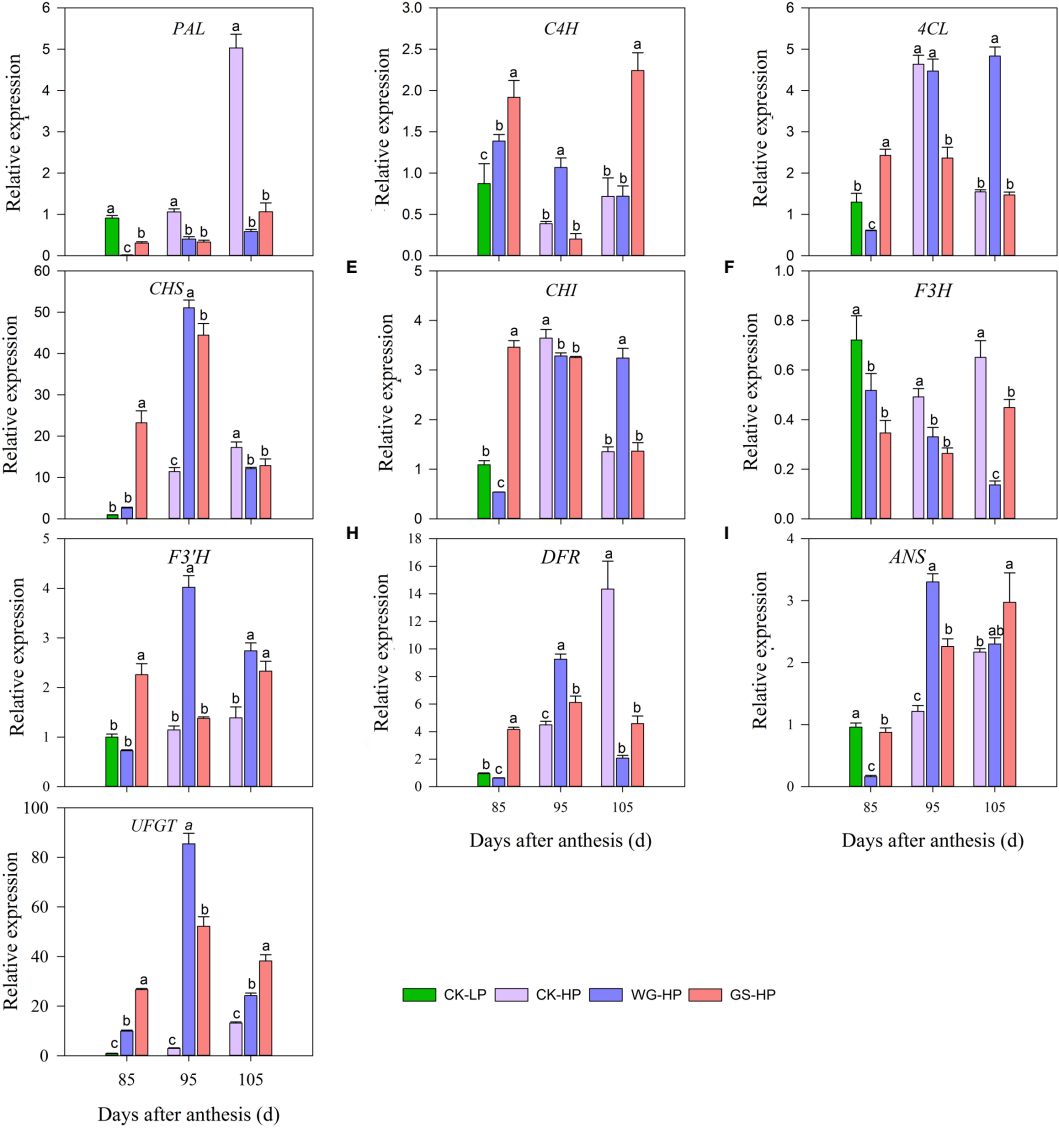
Transcript levels of anthocyanin-related genes in different treatments. CK-LP means green skin of ‘Yinhongli’plum, the rest are red skin. Values are mean ± SD (n =3). Different letters indicate significantly different values at *p*< 0.05. Phenylalanine ammonialyase, PAL **(A)**, namate-4-hydroxylase, C4H **(B)**, 4-coumaroyl: CoA-ligase, 4CL **(C)**, chalcone synthase, CHS **(D)**, chalcone isomerase, CHI **(E)**, flavanone 3-hydroxylase, F3H **(F)**, flavanone 3′-hydroxylase, F3’H **(G)**, dihydroflavonol 4-reductase, DFR **(H)** anthocyanidin synthase, ANS **(I)**; UDP-glucose: flavonoid 3-O-glucosyltransferase, UFGT **(J)**.

The expression levels of structural genes in anthocyanin metabolic pathways were further investigated in red skin of treatment WG, GS, and CK by qRT-PCR (**Figure 4**). At 85 DAA, the structural genes *phenylalanine ammonialyase* (*PAL*)*, namate-4-hydroxylase (C4H), 4-coumaroyl: CoA-ligase (4CL), chalcone synthase (CHS), chalcone isomerase (CHI), flavanone 3-hydroxylase (F3H), flavanone 3′-hydroxylase*(*F3’H*), *dihydroflavonol 4-reductase (DFR)* and *UDP-glucose: flavonoid 3-O-glucosyltransferase (UFGT)* in anthocyanin metabolic pathway of ‘Yinhongli’ plum treated by WG and GS were all expressed in red skin while CK was only expressed in the green skin. GS treatment had the highest expression level in most anthocyanin synthesis genes, such as *C4H, 4CL*, *CHS, CHI, F3’H, DFR*, and *UFGT*. While CK had the highest expression level of *F3H* and the lowest expression level of *UFGT*. At 95 DAA, fruit skin in treatment WG had the highest expression level of most genes, such as *C4H, CHS, F3’H, DFR*, and *ANS*. While at 105 DAA, the expression of anthocyanin structural genes *F3’H, anthocyanidin synthase(ANS)*, and *UFGT* were all up-regulated after treatment with biostimulant compared to untreated CK. Therefore, this led to an increase in anthocyanin accumulation in ‘Yinhongli’ plum. *UFGT* is a precursor enzyme of anthocyanin synthesis, which catalyzes the glycosylation of anthocyanin to form a stable anthocyanin-3-monoglycoside. At 85-105 DAA, the expression of structural gene *UFGT* was up-regulated after biostimulant application compared to control.

### 3.7 Correlation analysis between anthocyanins and sugars

Pearson correlation coefficients between anthocyanins and sugars calculated and listed in **Table 5**. In CK, the content of glucose and fructose showed positive correlation with the content of total anthocyanin. While for WG and GS treatments, sucrose and total sugar contents were positively correlated with total anthocyanin content. Sucrose and total anthocyanin content accumulated continuously during fruit ripening (**Figure 1D** and **Figure 2D**), which was consistent with the trend of skin color change (**Figure 1A**). In addition, the expression level of *PAL, CHS, DFR, ANS* and *UFGT* showed significant correlation with anthocyanin content (**Table 2**), *4CL* and *CHI* showed negative correlation with anthocyanin content, while only expression levels of *PAL* and ANS in GS treatment were highly significantly positively correlated with anthocyanin content.

**Table 5:** Correlation between the anthocyanins content and sugar content and gene expression level.

	Total anthocyanin content		
	CK	WG	GS
Glucose	0.945^**^	0.220	-0.925^**^
Fructose	0.929^**^	0.611	0.369
Sucrose	-0.245	0.946^**^	0.974^**^
Sorbitol	-0.603	-0.609	0.947^**^
Total sugar	0.749	0.790^*^	0.989^**^
*PAL*	0.979^**^	0.892^**^	0.816^**^
*C4H*	0.474	-0.914^**^	0.118
*4CL*	-0.952^**^	0.767^*^	-0.812^**^
*CHI*	-0.931^**^	0.710^*^	-0.887^**^
*CHS*	0.855^*^	-0.047	-0.290
*F3H*	0.663	-0.918^**^	0.483
*F3’H*	0.552	0.401	0.038
*DFR*	0.997^**^	-0.076	0.196
*ANS*	0.956^**^	0.475	0.936^**^
*UFGT*	0.948^**^	-0.014	0.396

** and * represent significant correlation at *P*<0.01 and *P*< 0.05, respectively.

## 4 Discussion

There was increasing evidence that biostimulant application increases single fruit weight, color, and sugar content (Annalisa et al., 2018; Mannino et al., 2020). In general, firmness, colour, sugar-acid ratio are considered important parameters for evaluating the quality of plums and determining their market value (Xu et al., 2020). Similar results were observed in our study. Principal component analysis (PCA) has been widely used for fruit quality evaluation of apples (Zhang et al., 2020), plums (Milošević and Milosevic, 2012), and other fruits (Finnegan and O’Beirne, 2015). The results of the PCA of 11 physiological indexes showed that the comprehensive quality of fruit of the biostimulation treatment was higher than that of the control (**Table 4**). In detail, Fruit weight (X1), TSS/TA (X3), a* (X5), cyanidin 3-*O*-glucoside (X6), cyanidin 3-*O*-rutinoside (X7), total anthocyanins (X8), Fructose (X10) and Sucrose (X12) reflected PC1, and the contribution rate accounted for 38.40% of the total variance. It can be used as the basis to evaluate the fruit flavor of ‘Yinhongli’ plum. a*(X5) dominated the third PC, representing 8.65% of the total variation. Similarly, the results of this study showed that Weiguo and Guanwu Shuang treatments significantly reduced L* and increased a* value compared to the control, which meant that fruitsdarker, redder and mature (Champa et al., 2015). In addition, the both treatments prompted the fruit to turn color earlier.

Carbohydrates are closely related to fruit quality and provide the carbon and energy basis for fruit taste, texture, flavor (Barrett et al., 2010). The type and content of soluble sugars affect fruit flavor (Guan et al., 2015), which is an important non-visual property for consumers (Awad and Jager, 2003; Giovannoni, 2004). Sucrose and sorbitol are important means of carbohydrate transport, moving from leaves to sink (fruits, roots, and stems) in the form of bast loading and converting to glucose and fructose. The intensity of sweetness depends on the specific sugar spectrum, and sucrose is the main contributor to sweetness (Cirilli et al., 2016). In this study, we found that the glucose and fructose contents of ‘Yinhongli’ plum fruit were high at 60-85 DAA (**Figure 3**). With the fruit ripening, sucrose accumulated, and the sucrose content was the highest at 105 DAA. Sorbitol is the main translocated soluble sugar in pear fruits (Mesa et al., 2016), including apple (Zhang et al., 2016), while sucrose is the main sugar transport in peach fruit (Aslam et al, 2019). Our resultst (**Figure 3D**) showed that ‘Yinhongli’ plum is sucrose accumulating, a result that is consistent with previous studies (Dugalic et al., 2014). Ren et al. (2021) found that a decrease in *AI* and *NI* activity during late fruit development resulted in lower sucrose catabolism and rapid sucrose accumulation. The present study is consistent with those of the above study results that *AI* and *NI* invertases transcript levels were higher at the initial stage of color conversion, which provided plentiful energy for fruit development. Therefore, changes in *AI* and *NI* transcript levels may play a major role in regulating soluble sugar metabolism.

The anthocyanin concentration and composition are important factors, which determined the color of plum fruits. The major anthocyanins in Japanese plum (*P. salicina*) fruit are cyanidin 3-galactoside (cy3-gal) and cyanidin 3-acetyl-glucoside (cy3-ace-glu) (Sahamishirazi et al, 2017), however, European plum (*Prunus domestica* L.) are mainly cyanidin 3-rutinoside (cy3-rut), peonidin 3-rutinoside (pe3-rut), 3-glucoside (cy3-glu), and peonidin 3-glucoside (pe3-glu) (Usenik et al., 2009). Our study demonstrated the plum cultivar, ‘Yinhongli’ plum, only contained two major anthocyanins: cyanidin 3-*O*-glucoside and cyanidin 3-*O*-rutinoside. These results are in agreement with previous results for ‘Taoxingli’ (*Prunus salicina* L.) varieties (Xu et al., 2020). And the variation trend of Cy3G was consistent with that of total anthocyanin, and no anthocyanin was detected in the green skin (**Figures 1D–F**). Anthocyanin belongs to flavonoids, the biosynthesis of its metabolites is regulated by some structural genes, and the accumulation of anthocyanins in fruit ultimately depends on the regulation of genes (Feng et al., 2010). Previous studies have reported that *CHS, F3H, DFR, ANS*, and *UFGT* are key genes for anthocyanin biosynthesis in fruits (Tsuda et al., 2004; Kim et al., 2005; Bashandy et al., 2015; Wu, et al., 2018). Similarly, this study found that the expression levels of anthocyanin structural genes increased with fruit maturation in the control treatment after fruit started to turn color, and *CHS, DFR, ANS*, and *UFGT* were highly significantly and positively correlated with total anthocyanin content (**Table 2**). Kobayashi et al. (2001) found that *UFGT* was only highly expressed in the skin of red varieties, whereas inhibition of *UFGT* gene transcriptional activity resulted in green skin (Wang et al., 2013). In this study, biostimulants increased the transcript levels of *CHS* and *UFGT* in the mid and latter stages of the skin of ‘Yinhongli’ plum fruits. Weiguo and Guangwu Shuang treatments promoted the rapid coloration of skin in the middle stage of color change, while the highest expression was observed in the Weiguo treatment at 105 DAA. This is likely due to anthocyanin synthesis-related genes, which were located upstream of anthocyanin synthesis, and biostimulants induce abundantly expression of the related genes, and the skin produces anthocyanin synthesis-related enzyme proteins. At this time, even if the upstream genes are no longer expressed in large amounts, the activity of the existing enzymes can maintain anthocyanin synthesis. However, at the same time, the large amount of anthocyanin synthesized may also feedback to inhibit the expression of the upstream genes, resulting in the peak of total anthocyanin content not coinciding with the period of peak gene expression. Studies have reported that seaweed and protein hydrolysate biostimulant products can enhance the intensity and area of skin coloration of ‘Jonathan’ apples at fruit ripening (Soppelsa et al., 2018). In the present study, biostimulant application significantly up-regulated the expression of structural genes in the anthocyanin synthesis pathway and also promoted the accumulation of total anthocyanin, which may be the cause of the poor skin coloration of CK-treated fruits. However, this did not change the two-sided color characteristics of the ‘Yinhongli’ plum fruit, which may be determined by genetic factors. Further studies are needed to validate this point.

In this study, we discovered that anthocyanin accumulation continues in the ‘Yinhongli’ plum started before the fruit ripened, and sugar accumulation lasted until the fruit ripened. soluble sugars are considered important signals for the regulate anthocyanin synthesis in addition to playing an important role in carbohydrate metabolism and fruit quality (Farcuh et al., 2018; Turkyilmaz et al., 2019). In our work, there was a significant correlation between total sugar and total anthocyanin contents (**Table 4**). Changes in carbohydrate and anthocyanin content during fruit ripening were associated with biostimulants on carbohydrate and anthocyanin-related gene expression. Studies have revealed that sucrose as an exogenous substance can regulate the expression of anthocyanin synthesis genes in the skin, and the expression of the structural anthocyanin genes *CHS, CHI, F3H, F3’H, DFR*, and *UFGT* were significantly up-regulated in Arabidopsis treated with exogenous sucrose, glucose and fructose (Boss et al., 1996; Sheng et al., 2005). Sugar induces the expression of genes related to anthocyanin synthesis and promotes anthocyanin synthesis (Meng et al., 2018). Sucrose accumulates continuously during fruit ripening of ‘Yinhongli’, which provides sufficient energy for sugar and anthocyanin metabolism (**Figures 2D**). In this study, We found that the transcript levels of most structural genes (*CHS, F3’H, ANS* and *UFGT*) in the anthocyanin synthesis pathway of the ‘Yinhongli’ plum were up-regulated after biostimulant treatment compared to the untreated control, which was consistent with the expression levels of genes related to sugar metabolism. This implies the importance of these genes in anthocyanin and sugar biosynthesis. Wu et al. (2018) found that 1-methylcyclopropene upregulated the transcript levels of *PAL, CHS, F3H* and *ANS*, and the corresponding anthocyanin content increased. Furthermore, this study found that biostimulant treatment significantly increased the accumulation of carbohydrates and anthocyanins in ‘Yinhongli’ plum fruits compared with CK treatment, which contributed to the early fruit turning color prematurely. These results pointed to the possibility that the accumulated sugars in the ‘Yinhongli’ plum play an important role in the induction of anthocyanin biosynthesis and the expression of regulatory genes, which are the leading causes of pigment formation in the skin of ‘Yinhongli’ plum.

## 5 Conclusion

Overall, the treatment of biostimulants promoted the accumulation of carbohydrates and improved the fruit coloration, and increased the nutritional value and marketability of ‘Yinhongli’ plum fruits. This did not change the two-sided color characteristics of the skin. Weiguo (Amino acid) and Guangwu Shuang (Amino acid) treatments significantly induced the up-regulation of gene expression of structural genes related to sugar metabolism and increased sugar content in fruit, while promoting the accumulation of anthocyanin in the skin. Biostimulant treatment induced high expression of the structural genes *CHS* and *UFGT* in the middle and late stages, which promoted the fruit color change of ‘Yinhongli’ plum in advance. These findings provide a direction for further studies on the interactions between anthocyanin and sugar metabolism synthesis pathways in the ‘Yinhongli’ plum.

## Data availability statement

Publicly available datasets were analyzed in this study. This data can be found here: https://blast.ncbi.nlm.nih.gov/Blast.

## Author contributions

Among the authors in the list, LY and YZP performed experiments, and data analysis and wrote the manuscript. DL and HX conducted the experiment design and revised the manuscript. QX, YH, WZ and CBP performed data analysis. JW and XLL conducted project Management and funding acquisition. All authors contributed to the article and approved the submitted version.
