# Supplementary material for: Biostimulants promote the accumulation of carbohydrates and biosynthesis of anthocyanins in ‘Yinhongli’ plum
Source: Front Plant Sci. 2023 Jan 6;13:1074965. doi: 10.3389/fpls.2022.1074965 (PMC9854126; doi:10.3389/fpls.2022.1074965)
Supplement: Supplementary file 1 [file Table_1.docx]

Supplementary Material

# Supplementary Tables

**Table S1** The primers’ sequences of anthocyanin and sugar selected genes

| Gene | Accession Number of Reference Genes | Sequence of primer（5′–3′） |
| --- | --- | --- |
| *PAL* | evm.model.Chr1.2407 | F:AGCACTTCAATCTTCCAA  R:GCACTTCCACTATCCAAT |
| *C4H* | evm.model.Chr1.365 | F:TCTTGAGAGGCTACTTGA  R:GCACTTCAGTCCTTCATT |
| *4CL* | evm.model.Chr8.333 | F:GGTTATCTTAATGATCCTGAGT  R:AAGCCTATATCGCCTGTAT |
| *CHI* | evm.model.Chr5.2237 | F:GAGTCCGTTGAGTTCTTC  R:TAGTGGCAGTATCGTTGT |
| *CHS* | evm.model.Chr2.5839_evm.model.Chr2.5843_evm.model.Chr2.5844 | F:GCCACAAGACACATACTA  R:CTTCCTTACCTCATCCAA |
| *F3H* | evm.model.Chr8.2031 | F:TACTACTCTCACCTCCAT  R:ATTGCTGAAGTTGTTGTA |
| *F3'H* | evm.model.Chr7.440 | F:AAGGCGGATGAGTTCAAG  R:GGGATAAAGTCACCTATGTTG |
| *DFR* | evm.model.Chr2.2057 | F:AAGAGCACCAGAAGTCAT  R:AAGTACATCCAACCAGTCA |
| *ANS* | evm.model.UTG5995.3 | F:ACTTCTTCCACCTTGTATA  R:GTAGCCTCAATGTAATCAG |
| *UFGT* 2 | evm.model.Chr5.3164 | F:TTCACAATCCAACAACTCACTCT  R:CGCCATCAGCCACATCAA |
| *AI* | evm.model.Chr7.1820 | F:AGACTGGAAGCAACCTAC  R:CCTTGACCTCTACCTTGT |
| *NI* | evm.model.Chr2.4895 | F:GTGGTGGATCATCTTGTTG  R:CCTTATGCCTGTCTGGAA |
| *SS* | evm.model.Chr6.2782 | F:TCACGGCTGACCTTATAG  R:GGCTCTCATACTGACCAA |
| *SPS* | evm.model.Chr8.2838 | F:GGATTAGTCAGCAGAAGG  R:CCACCAGTATCAGAGTCA |
| *SDH* | Pd.00g318450.m01.CDS | F:GTCTCAACAAACCCTCAG  R:CTCACATCTACTCCACCT |
| *GAPDH* | evm.model.Chr3.1223 | F:ACTTGTTCTTATGAGGAT  R:CATCTTCTTCAGTGTAAC |
